# Layout Comparison and Parameter Optimization of Supercritical Carbon Dioxide Coal-Fired Power Generation Systems under Environmental and Economic Objectives

**DOI:** 10.3390/e24081123

**Published:** 2022-08-15

**Authors:** Dongxu Chen, Zhonghe Han, Yaping Bai, Dongyang Guo, Linfei Zhao, Peng Li

**Affiliations:** 1 School of Energy Power and Mechanical Engineering, North China Electric Power University, Baoding 071003, China; 2 Hebei Key Laboratory of Low Carbon and High Efficiency Power Generation Technology, North China Electric Power University, Baoding 071003, China

**Keywords:** supercritical carbon dioxide, coal-fired power system, thermodynamic model, layout comparison, multi-objective optimization, decision-making

## Abstract

In the current studies, the supercritical carbon dioxide coal-fired power generation systems show efficiency and cost advantages over the traditional steam-based power systems. However, few studies have considered simultaneously environmental and economic objectives in the multi-objective analysis process. This study conducts a layout comparison and parameter optimization of the systems under the above two objectives. Initially, the thermodynamic, environmental, and economic models of the systems are established. Subsequently, the optimal layout is determined by the two-stage layout comparison. Further, multi-objective optimization is performed for the selected layout, and the optimal design parameters are determined by the decision process. Finally, the sensitivities of three selected parameters to the optimization results are analyzed. The results show that the basic layout coupled with overlap and intercooling schemes is optimal. Its ultimate environmental impact (*UEI*) and levelized cost of electricity (*LCOE*) are 219.8 kp-eq and 56.9 USD/MWh, respectively. The two objectives *UEI* and *LCOE* are conflicting. Based on a trade-off between them, the maximum temperature/pressure of the system is determined to be 635.3 °C/30.1 MPa. The coal price per unit of heat shows the highest sensitivity, and the pinch temperature difference of the recuperator shows opposite sensitivities at the *UEI* below 218 kp-eq and above 223 kp-eq.

## 1. Introduction

The reduction in environmental pollution has been resolutely considered worldwide. Clean and low carbon are the development trends in power generation systems. Renewable energy has attracted increasing attention [1], but it continues to have the disadvantage of intermittency [2]. According to the statistical data provided by British Petroleum (BP) [3], in 2021, the share of coal was 36% of the global power generation, which was still the dominant energy type. Therefore, the need for clean and efficient coal-fired power generation systems exists [4]. Currently, it is difficult to improve further the efficiency of traditional steam-based coal-fired power generation systems because of material limitations and the rapidly increasing costs of ultrahigh parameters [5]. The supercritical carbon dioxide (SCO_2_) Brayton cycle can solve this difficulty [6].

The SCO_2_ Brayton cycle was first proposed in the 1940s, but it did not receive significant attention until the 21st century owing to technical limitations [7]. Compared with other cycles, the biggest advantage of the SCO_2_ cycle is its high efficiency, which is closest to the efficiency limit of the Carnot cycle [6]. Moreover, it has other advantages, such as suitability for gas cooling [8], compact footprint [9], and decent flexibility [10]. In addition, the SCO_2_ cycle is suitable for different types of heat sources [11]. Its main application fields are nuclear, solar, fossil fuel, waste heat, and geothermal power [12]. At present, many studies on nuclear and solar power are available [13], whereas relatively few studies have focused on coal-fired power generation.

Some researchers have attempted its introduction into traditional steam-based coal-fired systems. Liu et al. [14] utilized the SCO_2_ cycle for the heat recovery of the tail flue gas of boilers. Xu et al. [15] attempted heating the SCO_2_ cycle using the 1–6 stage extracted steam from the turbines, and the waste heat of the SCO_2_ cycle was utilized to heat the air needed for combustion. Wang et al. [16] attempted heating the SCO_2_ cycle using the 3–5 stage extracted steam from the turbines, and the SCO_2_ cycle waste heat was utilized to heat the feedwater and air. Their results showed that the coupled system showed higher power generation efficiency and lower coal consumption rate.

However, more studies focus on the SCO_2_ coal-fired power generation (SCPG) systems. In these systems, the energy released by coal combustion is absorbed by the SCO_2_ cycle. Refs. [17,18], which studied the SCPG system, showed that it had an efficiency advantage over a traditional steam-based system. To further improve system efficiency, Refs. [18,19] adopted a method in which the bottom cycle was utilized to absorb the energy of tail flue gas. Moreover, Zhou et al. [20] analyzed SCPG systems with different capacities and concluded that the layouts of single reheat and double split flow were suitable for large-capacity systems. Bai et al. [21] introduced a spray attemperator into an SCPG system and found that the device could effectively adjust the working fluid temperature.

Currently, studies on the thermodynamic performance of SCPG systems are gradually maturing. In addition to thermodynamic performance, their economic performance should also be considered. The economic analysis of the SCPG system was performed in Refs. [22,23], where it was shown that it had a lower levelized cost of electricity (*LCOE*) than a traditional steam-based system. Xu et al. [24] adopted a more accurate economic model, indicating that the *LCOE* of the SCPG system was 1.32% lower than that of the traditional steam-based system and that the recuperator was the crucial component affecting the system costs. Sun et al. [25] compared different SCPG system layouts according to a unified standard and concluded that the basic cycle layout combined with a tail economizer, flue gas bypass, single-reheat, and intercooling had the best thermodynamic and economic performance. Moreover, Michalski et al. [26] compared different layouts under two indicators and found that the single recompression SCO_2_ cycle had the lowest break-even electricity price and the highest net efficiency. In Ref. [27], it was shown that the cost of electricity could not be reduced by increasing the turbine inlet temperature. In conclusion, it is commonly believed that the SCPG system has an economic advantage, whereas the options for superior parameters and layout improvement do not necessarily result in better economic performance.

In summary, the existing studies focus on the thermodynamic and economic performance of SCPG systems, whereas environmental protection has received increasing attention, and thus it is necessary to consider the environmental performance of these systems. The environmental impact load is an indicator that quantifies the impact degree of the system on the environment. Li et al. [28] adopted this indicator to compare an SCPG system with a traditional steam-based system. However, the single-objective analysis cannot reflect the comprehensive performance of the system, and thus multi-objective analysis is needed. For the multi-objective analysis considering environmental and economic performance, relevant studies are scarce. It is only found that Li et al. [28] conducted multi-objective optimization using the weighted summation method, but the comparison of different SCPG system layouts was not studied. Meanwhile, it lacks further analysis of multi-objective optimization results.

Based on the limitations of existing studies, the study of the layout comparison and parameter optimization of SCPG systems under environmental and economic objectives is conducted. The main purpose of this study is to determine the optimal layout and design parameters of the SCPG system by multi-objective analysis. Moreover, the novelties of this study are summarized as follows. First, the layout comparison considering environmental and economic objectives is performed. Second, comprehensive performance under environmental and economic objectives is regarded as the selection principle. Third, the characteristic and correlation analyses of multi-objective optimization results are conducted. Finally, the sensitivities of three selected parameters to the multi-objective optimization results are explored.

## 2. System Description

The layouts focused on in this study include typical and improved layouts. The distinction between typical layouts is the scheme of extracting tail flue gas energy. The distinction between improved layouts is the scheme of improving system efficiency.

### 2.1. Typical System Layouts

**Figure 1:**
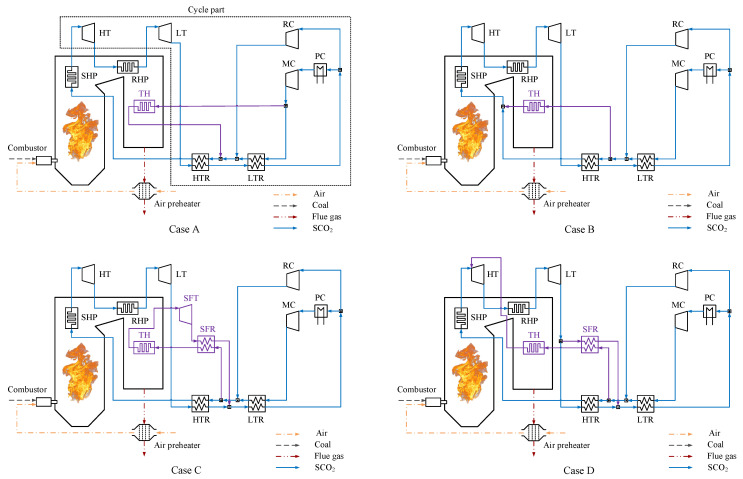
The basic layout coupled with four extraction schemes of tail flue gas energy: (**Case A**) LTR parallel scheme. (**Case B**) HTR parallel scheme. (**Case C**) Top-bottom scheme. (**Case D**) Overlap scheme.

Owing to the higher SCO_2_ temperature before entering the boiler, the temperature of the tail flue gas is higher. Therefore, the extraction of tail flue gas energy is one of the critical concerns of SCPG systems [29]. It is necessary to split the lower temperature SCO_2_ stream from the cycle part and utilize it to extract tail flue gas energy in the tail heater (TH). The basic SCPG system layout and its four extraction schemes constitute four typical system layouts, as shown in Figure 1. The components of the basic layout are shown in black. The extraction schemes are indicated in purple.

The basic layout consists of two parts: a boiler and a cycle. Specifically, the boiler includes heating surfaces, an air preheater, and a combustor. The heating surfaces consist of the superheat part (SHP) and reheat part (RHP). The SCO_2_ recompression cycle is adopted as the cycle part. It includes two compressors: a main compressor (MC) and a recompressor (RC), two turbines: a low-pressure turbine (LT) and a high-pressure turbine (HT), two recuperators: a low-temperature recuperator (LTR) and a high-temperature recuperator (HTR), and a precooler (PC). The cycle process is described as follows. The superheated SCO_2_ is expanded in the HT and is then sent to the RHP to be reheated. After being expanded in the LT, it enters the recuperation system to preheat the cold side stream and is then split into two streams. The main stream is allowed to enter the PC to release waste heat. After being compressed in the MC, it passes successively through the recuperation system and boiler to receive energy from the hot side stream and flue gas. The other stream is compressed in the RC, after which it is mixed into the cold side outlet of LTR.

Among the four extraction schemes, the reason behind choosing the former two is that they are representative heat absorption schemes using a direct split, whereas the reason for the latter two schemes is that they are representative heat absorption schemes based on a composite cycle. Their detailed explanations are as follows:

Case A (LTR parallel scheme): This scheme was adopted in Refs. [25,29]. The split path is parallel to the cold side of the LTR. Specifically, the stream is split from the outlet of the MC and mixed into the cold side outlet of the LTR.

Case B (HTR parallel scheme): This scheme was adopted in Refs. [25,30]. The split path is parallel to the cold side of the HTR. In particular, the stream is split from the cold side outlet of LTR and mixed into that of the HTR.

Case C (Top-bottom scheme): This scheme was proposed by Sun et al. according to the energy cascade utilization principle [31]. The stream is split from the cold side outlet of the LTR to form the bottom cycle to absorb the energy of the tail flue gas. The split flow turbine (SFT) and split flow recuperator (SFR) of the bottom cycle are independent of the top cycle. The heated stream is expanded in the SFT and is then mixed into the hot side outlet of the HTR after recuperation in the SFR.

Case D (Overlap scheme): This was proposed by Sun et al. according to the energy overlap utilization principle [32]. In contrast to Case C, the stream of the bottom cycle absorbs not only the energy of the tail flue gas but also the energy of the higher temperature flue gas. Therefore, the stream of the bottom cycle is heated to a higher temperature, and it shares turbines with the top cycle. The other parts are identical to those in Case C.

### 2.2. Improved System Layouts

**Figure 2:**
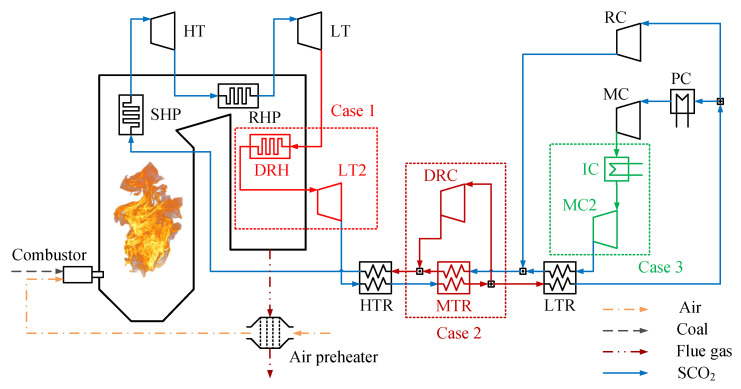
The basic layout coupled with three improved schemes: (**Case 1**) Double reheat scheme. (**Case 2**) Double recompression scheme. (**Case 3**) Intercooling scheme.

Based on the basic layout, three improved schemes are introduced to constitute three improved system layouts, as shown in Figure 2. The reason behind choosing them is that they are representative schemes for system efficiency improvement. These schemes target the expansion, recuperation, and compression processes of the system, respectively. The components of the basic layout are shown in black. The three improved schemes are indicated in other different colors. Their corresponding detailed explanations are as follows:

Case 1 (Double reheat scheme): The expanded stream in the LT is heated in a double reheater (DRH) and is then expanded in the LT2 to generate additional power. The average endothermic temperature of the SCO_2_ in the boiler is increased, and thus the system efficiency is increased compared with the basic layout.

Case 2 (Double recompression scheme): This scheme was first proposed by Moisseytsev [33]. Similar to the recompression scheme, a medium-temperature recuperator (MTR) is introduced. The stream of the hot side outlet of the MTR is split to be compressed in a double recompressor (DRC) and is then mixed into the cold side outlet of the MTR. The heat transfer temperature difference of the recuperation system is decreased, and thus the system efficiency is increased compared with the basic layout.

Case 3 (Intercooling scheme): The compressed stream in the MC is cooled in the intercooler (IC) and is then compressed in the MC2 to the maximum pressure of the system. The total power consumption of compressors is decreased, and thus the system efficiency is increased compared with the basic layout.

## 3. Methodology

**Figure 3:**
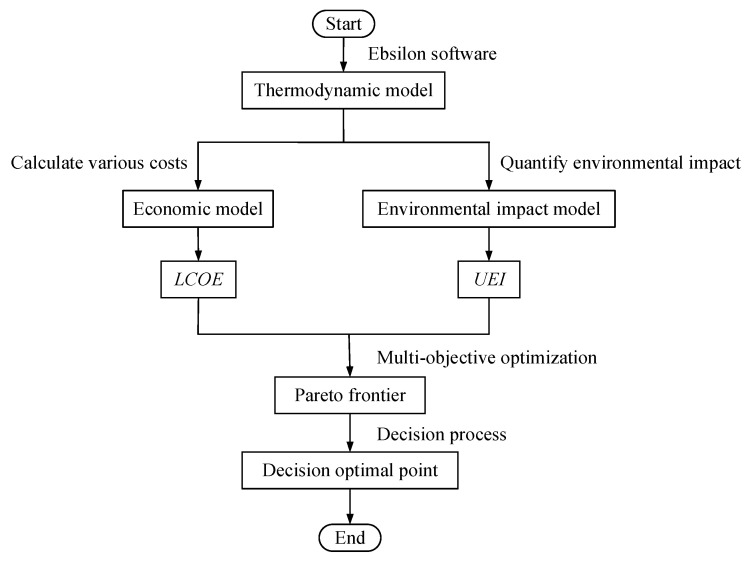
The logic flowchart of Section 3.

A logic flowchart of this section is shown in Figure 3. First, a thermodynamic model of the SCPG system is established using Ebsilon 13.02 software [34]. Second, based on this model, an economic and an environmental impact model are built by calculating various costs and quantifying the environmental impact of the system, respectively. The two objectives, namely, the levelized cost of electricity (*LCOE*) and the ultimate environmental impact (*UEI*) are obtained from the above models. Subsequently, multi-objective optimization for these two objectives is implemented to obtain the Pareto frontier. Finally, a decision process is conducted to find the decision optimal point from the Pareto frontier.

### 3.1. Thermodynamic Model

In this study, Ebsilon is employed to establish a thermodynamic model of the SCPG system. Because the corresponding codes are invisible to the users, it is necessary to verify the accuracy of this software. The simulation results of the entire system provided in Ref. [29] are used as a reference. In Ebsilon, the same model and input parameters as the above literature are applied. The comparison between present results and literature results is listed in Table 1. It can be seen that the errors are within a reasonable range, and thus the model established in Ebsilon is dependable.

In Ebsilon, the mass flow rate ($\dot{m}$), enthalpy (*h*), and composition of the flue gas are calculated according to the coal properties using an in-built function. This function can be expressed as Equation (1), the code of which is invisible to users.

(1) $${\dot{m}}_{\mathrm{fg}},h_{\mathrm{fg}},MF_{Y}=f(X_{\mathrm{ar}},\;\mathrm{LHV})$$

where *MF_Y_* is the mass fraction of matter *Y*; *X* represents the coal elements, such as C, H, O, and so on; LHV is the low heat value of the coal; subscript fg represents the flue gas; subscript *Y* represents the combustion products of coal, such as CO_2_, SO_2_, NO_x,_ and so on; subscript ar represents the as-received basis. The as-received basis is a benchmark, which stipulates selecting the actually received fuel when measuring its elemental composition. The properties of the selected coal samples are provided in Table 2.

Furthermore, the physical and thermodynamic properties of matters in Ebsilon are obtained from the standard reference database of the National Institute of Standards and Technology (NIST) [35]. Before establishing the system model, the following assumptions and considerations are formed.

1. The studied system is established as a steady state model.
2. The change of mechanical energy of working fluid is not considered.
3. The heat release from the cycle part to the environment can be neglected.
4. Except for the two streams at the outlet of the DRC and the cold side outlet of the MTR in Case 2, the two streams maintain identical temperatures before they are mixed [31].
5. For the boiler model, the exhaust flue gas loss and ash thermophysical loss are obtained from the simulated results. All other losses are set to 1.2% [36].
6. The pressure loss of the flue gas in the boiler is ignored [37].

The modeling process in Ebsilon involves selecting each component from the module library, connecting them using pipelines, setting input parameters, and performing simulations. The essence of the simulation is to solve the equation set generated from the energy equilibrium equations of all components. The energy equilibrium equations for the main components are presented in Table 3. The input parameters of the typical system models and those added owing to the improved schemes are listed in Table 4 and Table 5, respectively.

The system efficiency (*η*_sys_) is calculated as

(2) $$\eta_{\mathrm{sys}}=\frac{{\dot{W}}_{\mathrm{ele}}}{{\dot{m}}_{\mathrm{coal}}\cdot \mathrm{LHV}}$$

where $\dot{W}$_ele_ is the output electric power of the system.

### 3.2. Economic Model

In this study, the total revenue requirement (*TRR*) method [41] is applied to calculate the annual levelized economic costs of the SCPG system. The cost values are baselined to USD_2017_ by the chemical engineering plant cost index. The levelized total revenue requirement (*TRR_l_*) can be expressed as

(3) $$TRR_{l}=CC_{l}+OMC_{l}+FC_{l}$$

The levelized carrying charges (*CC_l_*) can be calculated as

(4) $$CC_{l}=TCI\cdot CRF$$

where *CRF* is the capital recovery factor.

The total capital investment (*TCI*) includes direct and indirect costs, which can be calculated according to the total purchased equipment cost (*PEC*_tot_).

(5) $$TCI=\psi \cdot PEC_{\mathrm{tot}}$$

where *ψ* is the relation coefficient between *TCI* and *PEC*_tot_.

The purchased equipment cost (*PEC*) of each component can be estimated by introducing the pressure correction coefficient (*f_p_*) based on the National Energy Technology Laboratory (NETL) method [42]. The core equation is as follows:

(6) $$PEC=a\cdot CP^{b}\cdot f_{t}\cdot f_{p}$$

where a and b are the fit coefficients according to the vendor quotes; *CP* is the characteristic parameter of the component; *f_t_* is the temperature correction coefficient. A detailed explanation for calculating the *PEC* for each component is provided in Ref. [43].

The levelized operating and maintenance costs (*OMC_l_*) and levelized fuel costs (*FC_l_*) are calculated as:

(7) $$OMC_{l}=OMC_{0}\cdot CELF$$

(8) $$FC_{l}=FC_{0}\cdot CELF$$

where *CELF* is the constant escalation levelization factor.

The calculation of the *CELF* is as follows:

(9) $$CELF=\frac{k(1-k^{n})}{1-k}CRF$$

(10) $$k=\frac{1+r_{n}}{1+i_{e}}$$

(11) $$CRF=\frac{i_{e}{\left(1+i_{e}\right)}^{n}}{{\left(1+i_{e}\right)}^{n}-1}$$

The first-year operating and maintenance costs (*OMC*) and fuel costs (*FC*) are calculated as [5]:

(12) $$OMC_{0}=\varphi_{\mathrm{fix}}\cdot TCI+\varphi_{\mathrm{var}}\cdot \tau \cdot {\dot{W}}_{\mathrm{ele}}$$

(13) $$FC_{0}={\dot{m}}_{\mathrm{coal}}\cdot \mathrm{LHV}\cdot \tau \cdot c_{\mathrm{coal}}$$

The values of economic parameters are listed in Table 6.

The levelized system costs (*SC_l_*) can be expressed as the sum of the levelized carrying charges and the levelized operating and maintenance costs.

(14) $$SC_{l}=CC_{l}+OMC_{l}$$

Finally, the *LCOE* is calculated as follows:

(15) $$LCOE=\frac{TRR_{l}}{\tau \cdot {\dot{W}}_{\mathrm{ele}}}$$

### 3.3. Environmental Impact Model

The calculation method for environmental impact adopted in the present study is a combination of the CML method [48] and the method proposed in Ref. [49]. The CML method is a method of assessing the impact of the system on the environment, which is developed by the Institute of Environmental Sciences of Leiden University. The considered environmental impact categories include global warming potential (GWP), acidification potential (AP), human toxicity potential (HTP), and dust pollution potential (DP). The pollutants considered in the system are CO_2_, SO_2_, NO_x,_ and dust. The removal efficiencies of SO_2_, NO_x_, and dust are 90% [50], 75% [51], and 99% [52], respectively.

The environmental impact of the *i*th category (*EI_i_*) can be calculated as

(16) $$EI_{i}=\sum_{j}AE_{j}\cdot CF_{j}$$

where *AE_j_* indicates annual emissions (kg/year) of the *j*th pollutant. *CF_j_* indicates the characterization factor of the *j*th pollutant, which is listed in Table 7. The unit of *EI* is the kilogram of pollutant equivalent per year (kg pollutant-eq/year).

The normalized environmental impact of the *i*th category (*NEI_i_*) can be calculated as

(17) $$NEI_{i}=EI_{i}/CEI_{90,i}$$

where *CEI*_90,*i*_ is the environmental impact per capita for China in 1990 of the *i*th category and is measured in kg pollutant-eq/year·p-eq. The unit of the *NEI* is the population equivalent (p-eq).

The ultimate environmental impact (*UEI*) can be calculated as

(18) $$UEI=\sum_{i}NEI_{i}\cdot WF_{i}$$

where the *WF_i_* is the weight factor of the *i*th environmental impact category.

The values of the environmental impact per capita for China in 1990 (*CEI*_90_) and the weight factor (*WF*) are obtained from Ref. [28] and are listed in Table 8.

### 3.4. Multi-Objective Optimization Method

There are two types of methods for solving multi-objective optimization problems: the weighted summation method and the Pareto frontier method [28]. The weighted summation method transforms a multi-objective problem into a single-objective problem using the weighted summation of each objective, whereas the Pareto frontier method uses Pareto improvement to allow multiple objectives to reach a state. In this state, one objective cannot be improved without worsening the others. The final result will obtain the Pareto optimal point set, which is called the Pareto frontier.

**Figure 4:**
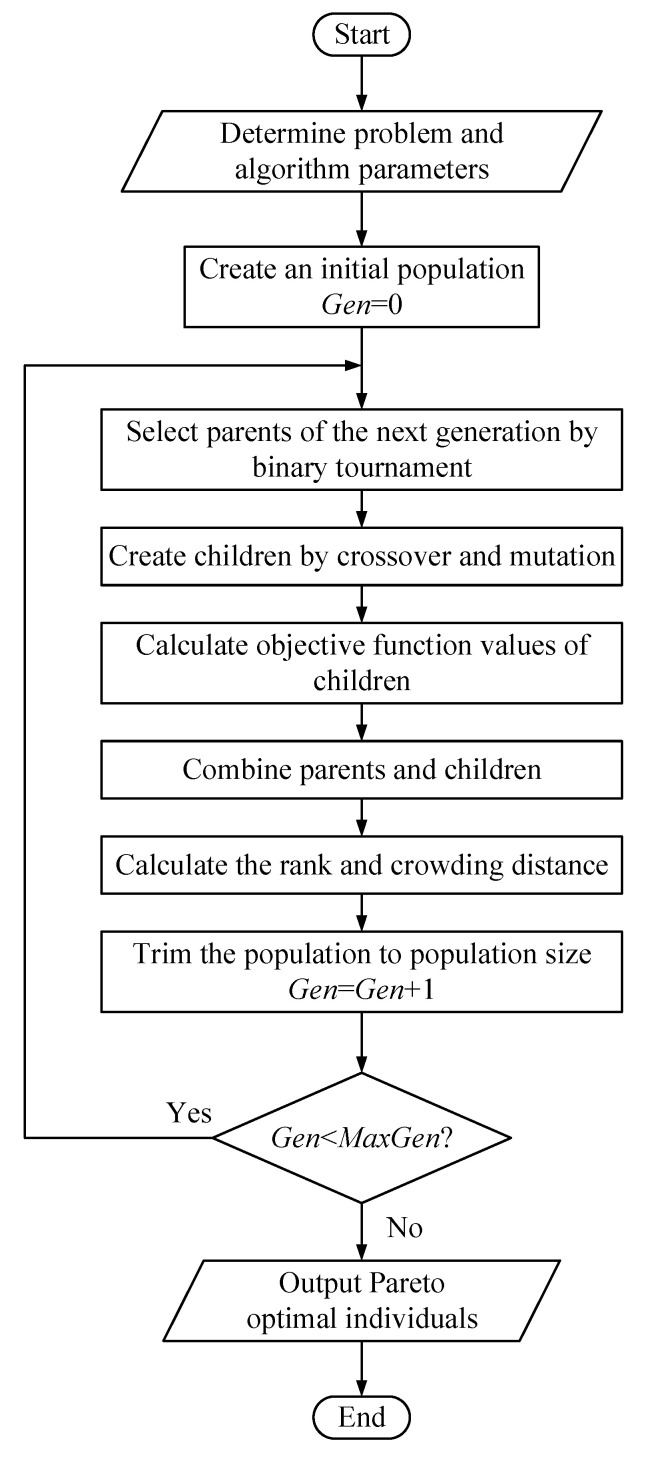
The flowchart of the gamultiobj algorithm.

The fast elitist non-dominated sorting genetic algorithm (NSGA-II) proposed by Deb et al. [54] belongs to the Pareto frontier method. In the present study, the gamultiobj algorithm in MATLAB R2014a software [55], which is a variant of NSGA-II, is adopted to perform multi-objective optimization. The biggest distinction between these two is the introduction of the Pareto fraction in the gamultiobj algorithm. This parameter is the ratio of the output Pareto optimal individuals to the population size. A flowchart of the gamultiobj algorithm is shown in Figure 4. First, the objectives, decision variables, and constraints of the problem are determined, and the algorithm parameters are set. Second, an initial population is created, the generation number (*Gen*) is marked as 0. Third, an iterative process is performed to achieve population evolution up to the maximum generation number (*MaxGen*), after which this iteration is stopped. Finally, the Pareto optimal individuals in the final population are obtained as the outputs. The values of the gamultiobj parameters in different sections are provided in Table 9.

The purpose of increasing system efficiency is to save costs and protect the environment. Therefore, the goal of this study is to simultaneously minimize *UEI* and *LCOE*. The calculations for the two objectives are expressed in Equations (18) and (15). Five design parameters of the system are selected as the decision variables. These are expressed as follows:

(19) $$\begin{array}{ll} \min. & \;UEI\left(t_{\max},p_{\max},p_{\mathrm{rh}},p_{\min},p_{\mathrm{ic}}\right)\;\&\\ & LCOE\left(t_{\max},p_{\max},p_{\mathrm{rh}},p_{\min},p_{\mathrm{ic}}\right) \end{array}$$

The range of decision variables and the corresponding constraints are as follows:

(20) $$\{\;\begin{array}{l} 500\;{}^{\circ}C<t_{\max}<700\;{}^{\circ}C\\ 22\;\mathrm{MPa}<p_{\max}<40\;\mathrm{MPa}\\ 12\;\mathrm{MPa}<p_{\mathrm{rh}}<21\;\mathrm{MPa}\\ 7.4\;\mathrm{MPa}<p_{\min}<10\;\mathrm{MPa}\\ 7.4\;\mathrm{MPa}<p_{\mathrm{ic}}<12\;\mathrm{MPa}\\ p_{\min}<p_{\mathrm{ic}} \end{array}$$

### 3.5. Decision Method

After obtaining the Pareto frontier, it is necessary to select a point from the Pareto frontier using a decision-making process. There are many decision methods, such as the ideal point method, principal component analysis method, and the analytic hierarchy process method. Among these, the ideal point method has the advantage of being simple and effective [56].

In this study, the technique for order preference by similarity to an ideal solution (TOPSIS) method proposed by Hwang and Yoon [57] is adopted. This method defines positive and negative ideal points, calculates their distances from each point, and ultimately searches for a point that is close to the positive ideal point and far from the negative ideal point. This point ensures a trade-off between multiple indicators and is called the decision optimal point (DOP). The steps of this method are detailed as follows [58].

For a decision-making problem, *m* candidates for competition and *n* indicators are involved in the evaluation, which can be represented as an *m* × *n* matrix, as shown in Equation (21).

(21) $$A={\left(a_{ij}\right)}_{m\times n}={\left[\begin{array}{cccc} a_{11} & a_{12} & \cdots & a_{1n}\\ a_{21} & a_{22} & \cdots & a_{2n}\\ \vdots & \vdots & \ddots & \vdots \\ a_{m1} & a_{m2} & \cdots & a_{mn} \end{array}\right]}_{m\times n}$$

where *a_ij_* represents the value of the *j*th indicator for the *i*th candidate. Hereafter, *i* = 1, 2, …, *m*; *j* = 1, 2, …, *n*.

First, a normalization from matrix ***A*** to matrix ***B*** is conducted to eliminate the dimensional effect of the different indicators, as shown in Equation (22).

(22) $$B={\left(b_{ij}\right)}_{m\times n}\;,\;b_{ij}=\frac{a_{ij}}{\sqrt{\;\sum_{i=1}^{m}a_{ij}{}^{2}}}$$

where *b_ij_* represents the dimensionless value of the *j*th indicator for the *i*th candidate.

Second, the positive and negative ideal points are confirmed. The positive ideal point ($c_{j}^{+}$) is where each indicator reaches the maximum (for benefit attribute) or minimum (for cost attribute) of all points. The negative ideal point ($c_{j}^{-}$) is where each indicator reaches the minimum (for benefit attribute) or maximum (for cost attribute) of all points. These two points are expressed as follows:

(23) $$c_{j}^{+}=\{\begin{array}{l} \underset{i}{\max}b_{ij}\;\mathrm{if}\;j\;\mathrm{is}\;\mathrm{benefit}\;\mathrm{attribute}\\ \underset{i}{\min}b_{ij}\;\mathrm{if}\;j\;\mathrm{is}\;\cos t\;\mathrm{attribute} \end{array}$$

(24) $$c_{j}^{-}=\{\begin{array}{l} \underset{i}{\min}b_{ij}\;\mathrm{if}\;j\;\mathrm{is}\;\mathrm{benefit}\;\mathrm{attribute}\\ \underset{i}{\max}b_{ij}\;\mathrm{if}\;j\;\mathrm{is}\;\cos t\;\mathrm{attribute} \end{array}$$

Third, in the *n*-*dimension* space, the Euclidean distance between each point and the positive ideal point ($d_{i}^{+}$) and that between each point and the negative ideal point ($d_{i}^{-}$) are calculated as follows:

(25) $$d_{i}{}^{+}=\sqrt{\;\sum_{j=1}^{n}{\left(b_{ij}-c_{j}{}^{+}\right)}^{2}}$$

(26) $$d_{i}{}^{-}=\sqrt{\;\sum_{j=1}^{n}{\left(b_{ij}-c_{j}{}^{-}\right)}^{2}}$$

**Figure 5:**
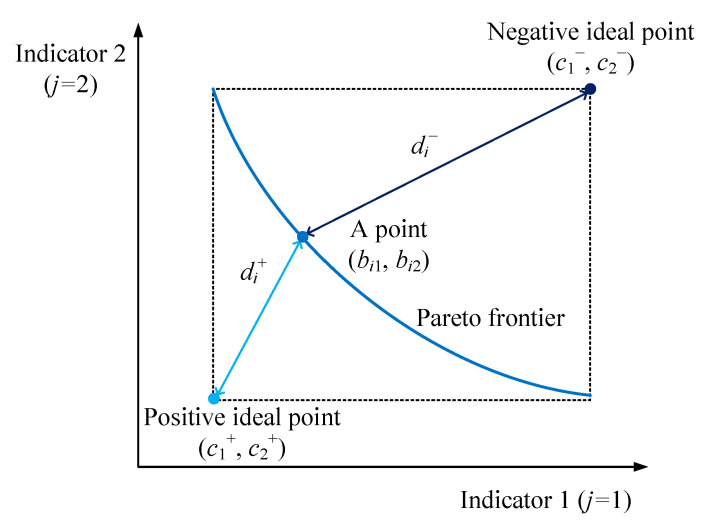
The diagram of the second and third steps of the TOPSIS method.

For a graphical display, only two indicators with cost attributes are assumed. Based on this assumption, a diagram of the second and third steps of the TOPSIS method is shown in Figure 5.

Fourth, the closeness coefficient (*CLC_i_*) is used to weigh the relative distance between each point and the two ideal points, which can reflect the degree of relative closeness to the positive ideal point, and is expressed as follows:

(27) $$CLC_{i}=\frac{d_{i}{}^{-}}{d_{i}{}^{+}+d_{i}{}^{-}}$$

Finally, all points are sorted in descending order according to the *CLC_i_*. The point with the maximum *CLC_i_* is considered as the decision optimal point.

## 4. Results and Discussion

### 4.1. Comparison of Different Layouts

In this section, under the objectives of *UEI* and *LCOE*, the comparisons of four typical layouts and three improved layouts are conducted to select the optimal layout with the best comprehensive performance.

#### 4.1.1. Comparison of Typical Layouts

**Figure 6:**
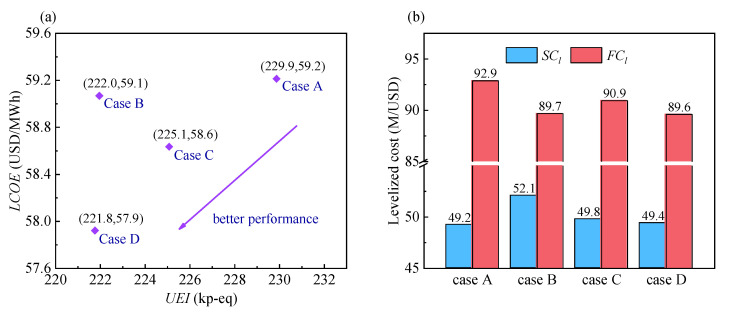
Comparison of four typical layouts: (**a**) *UEI* and *LCOE*. (**b**) levelized system costs (*SC_l_*) and levelized fuel costs (*FC_l_*).

Figure 6a shows a comparison of the *UEI* and *LCOE* for the four typical layouts. Both objectives are cost attributes, and thus the closer a point is to the bottom left, the better the comprehensive performance it provides. Comparisons of the thermodynamic and economic parameters for the four typical layouts are shown in Figure 6b and Table 10.

From Figure 6a, it can be observed that Case D has the lowest *UEI* and *LCOE*. This is because it has the highest *η*_sys_ and the second-lowest *SC_l_*. The reason for its highest *η*_sys_ is that it has the highest heat absorption quantity of tail flue gas. Case A has the highest *UEI* and *LCOE* values. This is mainly because it has the lowest *η*_sys_, which leads to the highest *FC_l_*. The reason for its lowest *η*_sys_ is that it has the lowest heat absorption quantity of tail flue gas. Although *SC_l_* is the lowest, the *LCOE* is the highest owing to its large *FC_l_*.

Cases B and C have different advantages. Compared with Case C, Case B has a lower *UEI*. This is because its *η*_sys_ is higher than that of Case C, which is consistent with the result in Ref. [25]. Compared with Case B, Case C has a lower *LCOE* mainly because of the drastic decrease in the HTR cost, which reduces its *SC_l_*. Specifically, the introduction of the SFR shares the huge heat transfer of the HTR, which increases the heat transfer temperature difference, ultimately causing a drastic decrease in the HTR cost. Compared with Case C, the *UEI* of Case B decreases by 1.39% at the expense of increasing the *LCOE* by 0.74%. The benefit outweighs the expense. Hence, the comprehensive performance of Case B is better than that of Case C.

In conclusion, the comprehensive performance ranks of the typical layouts in descending order are Case D, Case B, Case C, and Case A. Therefore, Case D is selected as the system to be improved upon in the next section. Moreover, in the comparison of Cases B and D, the introduction of SFR increases the heat transfer temperature difference, and the *SC_l_* is reduced while the *η*_sys_ is increased. Therefore, it is feasible to increase the heat transfer temperature difference by introducing an SFR.

#### 4.1.2. Comparison of Improved Layouts

**Figure 7:**
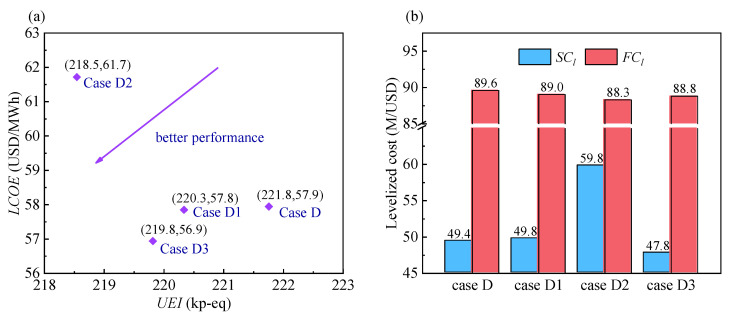
Comparison of three improved layouts: (**a**) *UEI* and *LCOE*. (**b**) levelized system costs (*SC_l_*) and levelized fuel costs (*FC_l_*).

Figure 7a compares the *UEI* and *LCOE* for the three improved layouts. Comparisons of the thermodynamic and economic parameters for these layouts are presented in Figure 7b and Table 11.

In comparison with Case D, the *UEI* and *LCOE* are reduced in Cases D1 and D3. Furthermore, the reductions in Case D3 are more prominent than those in Case D1. In particular, in Case D1, the double reheat process increases the average endothermic temperature of SCO_2_, the *η*_sys_ is improved, and the *UEI* is ultimately reduced. The introduction of LT2 increases the *SC_l_*. In Case D3, the intercooling process decreases the MC power consumption. The coal consumption decreases due to a constant output of electric power, which consequently decreases the *UEI*. Despite the increased number of components, the heat transfer quantity of the LTR is decreased, and the LTR cost is reduced. As a result, the *SC_l_* is reduced.

For Case D2, the second split process reduces the mass flow rate of exothermic SCO_2_, which consequently reduces the total heat release quantity in the PC. Meanwhile, the heat transfer temperature difference of the recuperation system decreases owing to the double recompression process. However, the corresponding costs of the recuperation system increase. Compared with Case D, the *UEI* decreases by 1.45% at the expense of increasing the *LCOE* by 6.55%. In other words, it pays high expense while gaining low benefit. Hence, the comprehensive performance of Case D2 is worse than that of Case D, which also implies that the heat transfer temperature difference of the regenerator is not necessarily the smaller the better.

In conclusion, the comprehensive performance ranks of the improved layouts in descending order are Case D3, Case D1, Case D, and Case D2. Therefore, Case D3 is the optimal layout and is selected as the system to be optimized in the subsequent section.

### 4.2. Analysis of Multi-Objective Optimization Results

Multi-objective optimization is implemented to obtain the Pareto frontier of the layout of Case D3. Subsequently, the characteristics and correlations of all the design parameter points in the Pareto frontier are explored.

#### 4.2.1. Evolution Process of Pareto Frontier

**Figure 8:**
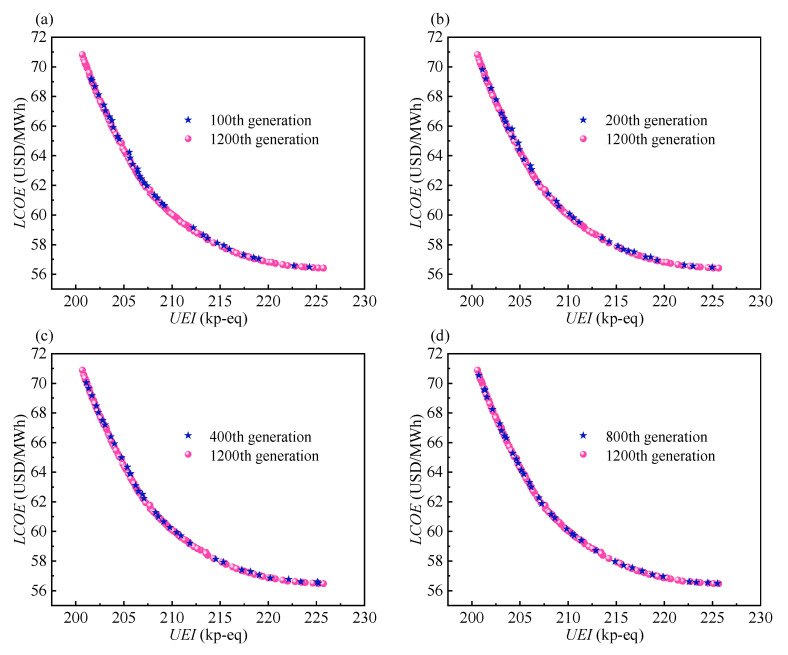
The Pareto frontier of different generations: (**a**) 100th generation. (**b**) 200th generation. (**c**) 400th generation. (**d**) 800th generation.

Figure 8 shows the Pareto frontier with the objectives of the *UEI* and *LCOE* for the different generations. The Pareto frontier of the 1200th generation is the final result and is used as a reference for other generations to reflect its evolution process. The reason for the higher number of points in the 1200th generation is the increased Pareto fraction after the 1000th generation.

From Figure 8, it can be observed that the Pareto frontier of the 100th generation is close to that of the 1200th generation, which means that the evolution process is fast. As the number of generations increases further, the two endpoints of the Pareto frontier appear in the trend of the extension. From the 100th to the 200th generation, the speed of extension is fast, after which it slows down. By the 200th generation, the two endpoints are close to the final endpoints of the 1200th generation. Hence, the 200th generation reflects the profile of the 1200th generation, which explains why the *MaxGen* is set to 200 in the sensitivity analysis. Moreover, the final result indicates that the two objectives conflict with each other. In other words, the benefit of one objective comes at the expense of the other. This indicates that each point is a candidate, and thus it is necessary to perform a characteristic analysis for all points.

#### 4.2.2. Characteristic Analysis of Pareto Optimal Points

**Figure 9:**
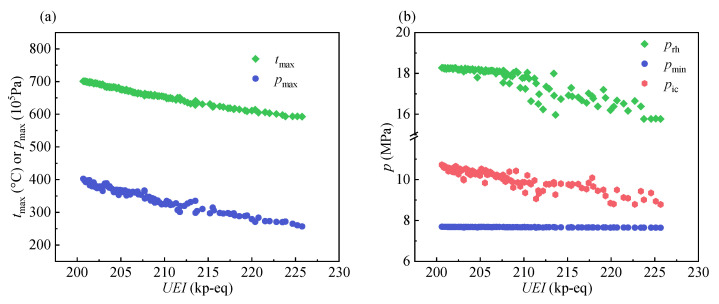
The variation of design parameters: (**a**) *t*_max_ and *p*_max_. (**b**) *p*_rh_, *p*_min,_ and *p*_ic_.

**Figure 10:**
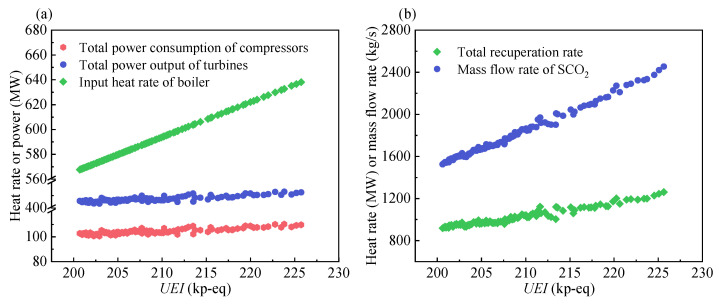
The variation of system output parameters: (**a**) input heat rate of boiler, total power output of turbines, and the total power consumption of compressors. (**b**) total recuperation rate and mass flow rate of SCO_2_.

In this section, the variations of the thermodynamic and economic parameters corresponding to the Pareto optimal points are presented to reflect the characteristics of all Pareto optimal points. The variations of the design parameters and system output parameters corresponding to the Pareto optimal points are shown in Figure 9 and Figure 10, respectively. The design parameters include the maximum temperature of the system (*t*_max_), the maximum pressure of the system (*p*_max_), the reheat pressure (*p*_rh_), the minimum pressure of the system (*p*_min_), and the inlet pressure of the intercooler (*p*_ic_).

It can be seen from Figure 9a that the *t*_max_ and *p*_max_ tend to decrease with an increase in the *UEI*. It can be explained that high *UEI* values indicate that high efficiency is no longer needed. As a result, the maximum temperature/pressure of the system decreases. As shown in Figure 9b, the two pressure parameters *p*_rh_ and *p*_ic_ tend to decrease because of the decrease in the *p*_max_. It is worth mentioning that the points of the *p*_rh_ and *p*_ic_ are concentrated and show an approximately linear decrease when the *UEI* is below 207 kp-eq. Above this value, their points become dispersed, which indicates that the correlations between them and the *UEI* decrease.

As shown in Figure 10a, the input heat rate of the boiler shows the largest variation with an increase in the *UEI*. The variations in the total power output of the turbines and the total power consumption of the compressors are identical because the output electric power of the system is set to a constant value of 300 MW. Referring to Figure 10b, the total recuperation rate and mass flow rate of SCO_2_ increase with the increasing *UEI*. This can be explained by the fact that an increase in the input heat rate of the boiler requires more SCO_2_ to absorb heat. Moreover, an increase in the mass flow rate leads to an increase in the total recuperation rate.

**Figure 11:**
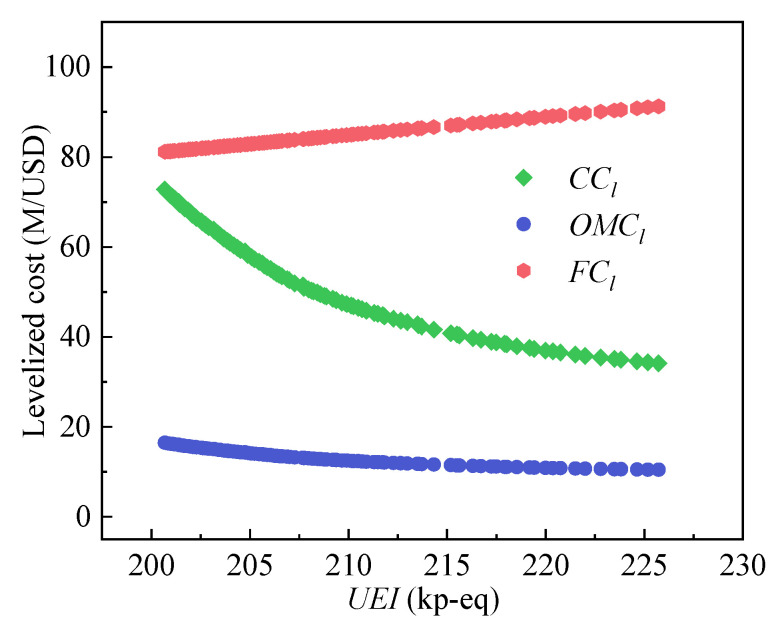
The variation of levelized costs.

Figure 11 plots the variation of carrying charges (*CC_l_*), operating and maintenance costs (*OMC_l_*), and fuel costs (*FC_l_*) corresponding to the Pareto optimal points. It can be seen that *CC_l_* decreases with the increase in the *UEI* and that the curve tends to flatten gradually. Moreover, the *FC_l_* values are larger than the *CC_l_* values among all the *UEI* regions, while the variation of *FC_l_* is less than that of *CC_l_*. With the decrease in the *UEI*, the *t*_max_ increases. More expensive materials are needed to resist high temperatures, and thus the *CC_l_* increases. An increase in the *t*_max_ will increase the *η*_sys_ and reduce the *FC_l_*, but the increase in material costs is more prominent. Therefore, the variation of *CC_l_* is larger than that of *FC_l_*. In addition, the profile of *CC_l_* is similar to that of the *LCOE*, which indicates that the variation of *CC_l_* accounts for the main influence on the *LCOE*. Therefore, more attention should be focused on it.

**Figure 12:**
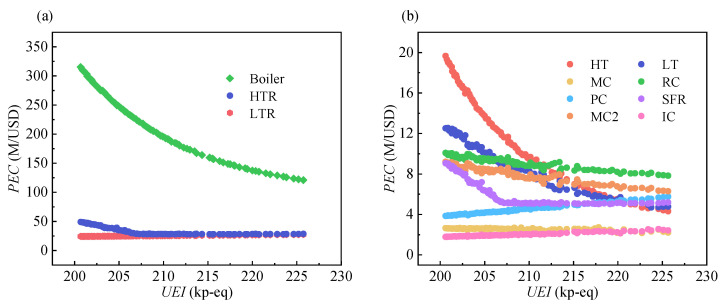
The variation of *PEC*: (**a**) *PEC* over 20 M/USD. (**b**) *PEC* below 20 M/USD.

Figure 12 displays the variation of purchased equipment cost (*PEC*) corresponding to Pareto optimal points, which is divided into two figures depending on whether the *PEC* is larger than 20 M/USD. As shown in Figure 12a, the *PEC* of the boiler shows the largest values and largest variation among the three components. Moreover, the profile of boiler *PEC* is similar to the profile of *CC_l_*. These results indicate that the boiler *PEC* accounts for the main influence on *CC_l_*. The variation of the boiler *PEC* is more apparent in the lower *UEI* region. This implies that as the *UEI* decreases, a higher boiler cost is required for reducing an identical *UEI*. Moreover, a *UEI* of 207 kp-eq splits the curve of the HTR into two parts. In the right part, the HTR *PEC* increases gently, whereas, in the left part, it shows a relatively rapid decline. A similar trend is visible in the curve of SFR *PEC*, as shown in Figure 12b. The reason for these trends is as follows: With the decrease in the *UEI*, the *η*_sys_ increases, the heat transfer quantity of these two components decreases, and thus the *PEC* decreases. When the *UEI* decreases below 207 kp-eq, the increase in the material costs caused by higher temperature becomes more prominent, and thus the *PEC* increases rapidly.

#### 4.2.3. Correlation Analysis of Pareto Optimal Points

According to the analysis of Figure 9, a certain relation exists between the design parameters and the objective. Therefore, as proposed by Spearman in 1904, the Spearman correlation coefficient (*ρ*_s_) [59], which could take values in the range of −1 and 1, is applied to quantify the relation. Here, positive and negative values denote positive and negative correlations, respectively. The larger the absolute value, the stronger the correlation.

Table 12 presents the *ρ*_s_ among the seven parameters (five design parameters and two objectives), which are sorted by absolute values in descending order. It can be seen that the *ρ*_s_ between the *UEI* and *LCOE* is −1, which indicates their completely monotonic negative correlation. The *t*_max_ and *p*_max_ show the 2nd and 4th strongest correlations, respectively, with the two objectives. This indicates that the *t*_max_ and *p*_max_ are the first and second most crucial parameters of the system, respectively. The *t*_max_ and *p*_max_ show the 6th strongest correlation with each other, and thus coordination between them is required in the parameter design process. In other words, their design values are determined together according to the relation between them.

**Figure 13:**
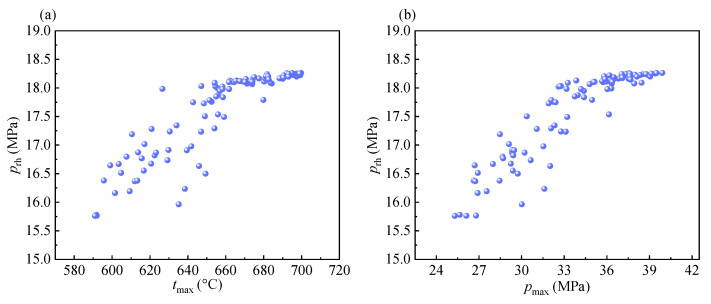
The relation between two parameters: (**a**) *p*_rh_ and *t*_max_. (**b**) *p*_rh_ and *p*_max_.

Furthermore, relatively weaker correlations are observed between the *p*_rh_ and the two objectives, *p*_rh_ and *t*_max_, and between the *p*_rh_ and *p*_max_, which rank 7th, 9th, and 10th, respectively. These indicate that the *p*_rh_ is the third most crucial parameter of the system. In Figure 13, the relation between the *p*_rh_ and *t*_max_, and that between *p*_rh_ and *p*_max_ are plotted as a scatter plot to visualize these relations. It can be observed that close correlations appear in the high-parameter region.

### 4.3. Comparison of Three Optimal Points

**Figure 14:**
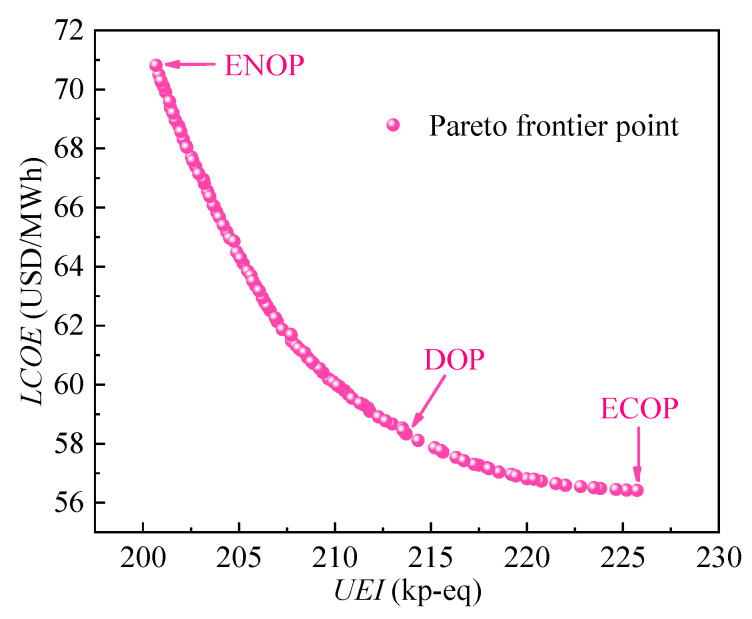
Three optimal points in the Pareto frontier.

The decision optimal point (DOP) is obtained through the decision process, which is then compared with the environmental optimal point (ENOP) and economic optimal point (ECOP) to demonstrate its advantage. As shown in Figure 14, these three optimal points are marked in the Pareto frontier. It can be observed that a huge expense of *UEI* is required to reduce the *LCOE* around the ECOP. Moreover, the *LCOE* expense of reducing the *UEI* is high around the ENOP. The DOP lies between the ENOP and ECOP, which is a trade-off between the two objectives. The advantage of the DOP is that it simultaneously maintains lower environmental impact and economic costs.

The values of the five design parameters and two objectives corresponding to the three optimal points are listed in Table 13. It can be seen that the maximum temperature/pressure of the system is 591 °C/25.4 MPa when the *LCOE* is the lowest. Moreover, the *t*_max_ and *p*_max_ reach the maximum values of the given boundary when the *UEI* is the lowest. For DOP, the maximum temperature/pressure of the system is found to be 635.3 °C/30.1 MPa. Compared with the ECOP, the *UEI* decreases by 5.3% at the expense of increasing the *LCOE* by 3.4%. Compared with the ENOP, the *LCOE* decreases by 17.7% at the expense of increasing the *UEI* by 6.5%. In other words, high benefits could be obtained at low expenses, and thus the DOP has a better comprehensive performance.

**Figure 15:**
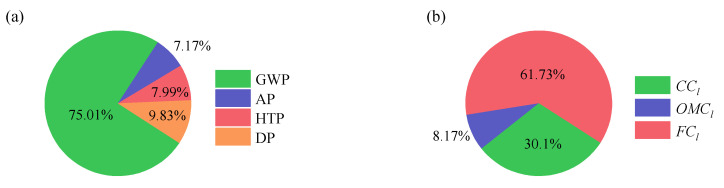
The ingredient distribution: (**a**) *UEI*. (**b**) *LCOE*.

For further analysis of the DOP, the ingredient distributions of the *UEI* and *LCOE* are shown in Figure 15. The ingredient distributions of *UEI* include global warming potential (GWP), acidification potential (AP), human toxicity potential (HTP), and dust pollution potential (DP). As shown in Figure 15a, GWP contributes approximately 75% of the *UEI*, which is due to the massive emissions of CO_2_ without the capture process. Therefore, the challenge of the SCPG system is to decrease CO_2_ emissions. The second contribution is DP, which accounts for 9.83% of the *UEI*. Although the dust removal efficiency has reached 99%, its environmental impact continues to be higher than that of SO_2_ and NO_x_. Hence, dust emissions should also be considered seriously. Moreover, as shown in Figure 15b, *FC_l_* accounts for more than half of the *LCOE* (61.73%), followed by *CC_l_* (30.1%) and *OMC_l_* (8.17%).

**Figure 16:**
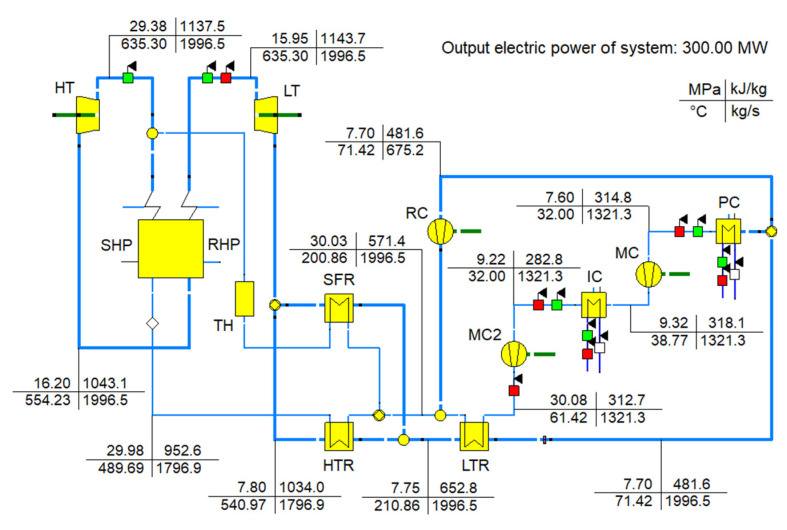
The Ebsilon model of the system with Case D3 layout and DOP parameters.

Finally, the basic layout coupled with overlap and intercooling schemes (Case D3) is determined to be the optimal layout. The decision optimal point (DOP) is determined to be the optimal design parameters. The Ebsilon model of this system is demonstrated in Figure 16.

### 4.4. Sensitivity Analysis

**Figure 17:**
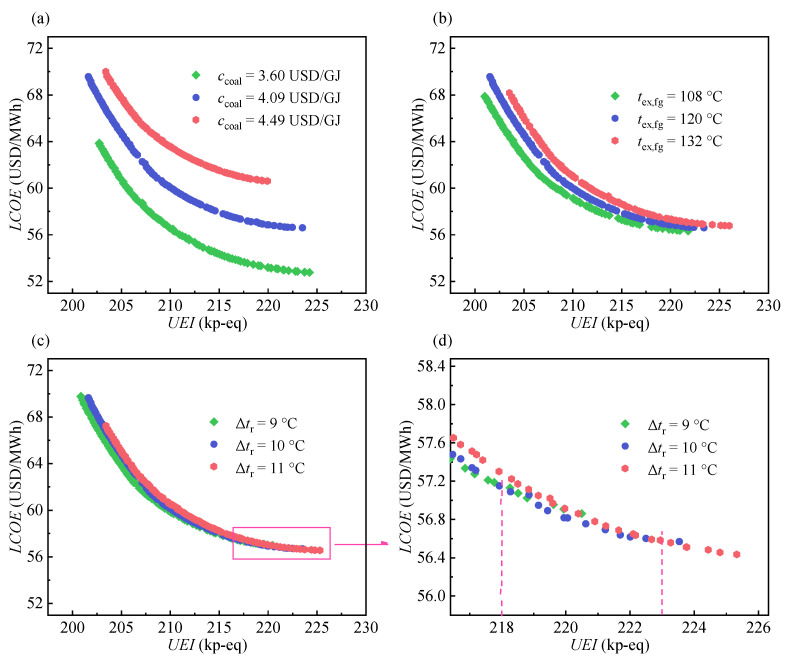
Sensitivity analysis for different parameters: (**a**) coal price per unit of heat. (**b**) exhaust temperature of flue gas. (**c**,**d**) pinch temperature difference of recuperator.

A sensitivity analysis is conducted to explore the effect of the coal price per unit of heat (*c*_coal_), exhaust temperature of flue gas (*t*_ex,fg_), and the pinch temperature difference of the recuperator (Δ*t*_r_) on the Pareto frontier. Figure 17 displays the results with a ±10% variation in these three parameters. Figure 17d is the partial enlargement of Figure 17c. Based on observations and comparisons, the following three points are worth mentioning.

First, the *c*_coal_ shows the highest sensitivity, which is because the *c*_coal_ directly affects the *FC_l_*. The *t*_ex,fg_ directly affects the boiler efficiency, further indirectly affecting the *SC_l_* and *FC_l_*. Hence, it has the second highest sensitivity. The Δ*t*_r_ has the least relation with the *SC_l_* and *FC_l_* than others, and thus its sensitivity is the lowest.

Second, the distance between different the Pareto frontiers gradually increases with an increasing *UEI* in the case of the *c*_coal_, which indicates that the sensitivity of *c*_coal_ to *LCOE* gradually increases with increasing *UEI*. This can be explained by the fact that in the high *UEI* region, the share of the *FC_l_* to the total costs is larger than that in the low *UEI* region. Because *c*_coal_ is directly related to *FC_l_*, high sensitivity appears in the high *UEI* region. In contrast, the sensitivities of *t*_ex,fg_ and Δ*t*_r_ to *LCOE* gradually decrease with the increase in *UEI*.

Third, as shown in Figure 17d, the curves of the Pareto frontier intersect in the high *UEI* region in the case of Δ*t*_r_. When the *UEI* is below 218 kp-eq, the *LCOE* decreases with a decrease in the Δ*t*_r_ for the same value of the *UEI*. In contrast, the *LCOE* increases when the *UEI* exceeds 223 kp-eq. For the former case, at the same *UEI*, the *t*_max_ and *p*_max_ will decrease with a decreasing Δ*t*_r_. Because the resultant cost reduction exceeds the increase in the recuperator cost, the *LCOE* is ultimately reduced. For the latter case, the resultant cost reduction is insufficient to offset the increasing recuperator cost, thus resulting in an increased *LCOE*.

## 5. Conclusions

In this study, environmental and economic objectives are considered to conduct layout comparison and parameter optimization of the SCO_2_ coal-fired power generation system. Specifically, four typical and three improved layouts are compared to select the optimal layout. Subsequently, multi-objective optimization is performed to obtain the Pareto frontier of the selected layout. Further, for the Pareto frontier, characteristic and correlation analyses, decision process, and sensitivity analysis are successively conducted. The following conclusions are drawn.

1. Overlap is the optimal scheme for the extraction of tail flue gas energy, and intercooling is the optimal improved scheme. Case D3 is the optimal layout with the ultimate environmental impact (*UEI*) of 219.8 kp-eq and levelized cost of electricity (*LCOE*) of 56.9 USD/MWh.
2. The two objectives, namely, *UEI* and *LCOE,* conflict with each other. The Spearman correlation coefficient between the maximum temperature and pressure of the system is 0.966, which indicates that a coordination between them is required in the parameter design process.
3. The decision optimal point shows a better comprehensive performance, the maximum temperature/pressure of which is 635.3 °C/30.1 MPa. Compared with economic and environmental optimal points, it takes 3.4% and 6.5% expenses in exchange for 5.3% and 17.7% benefits.
4. The coal price per unit of heat shows the highest sensitivity and the sensitivity of it to the *LCOE* is higher in the higher *UEI* region. The pinch temperature difference of recuperator shows opposite sensitivities when the *UEI* is below 218 kp-eq and above 223 kp-eq.

At present, the concept of SCO_2_ coal-fired power generation system is in the transition stage from theoretical study to engineering application. This study could provide some reference for the layout selection and parameter design of real systems in the future.

## Figures and Tables

**Table 1:** The comparison between present results and literature results.

Items	Literature Results	Present Results	Errors
**Cycle part**			
Heat transfer of recuperator (MW)	3822.59	3821.73	−0.02%
Power output of turbine (MW)	1359.81	1355.53	−0.31%
Power consumption of compressor (MW)	359.81	355.53	−1.19%
Heat release of cooler (MW)	952.51	949.63	−0.30%
Efficiency of cycle (%)	51.22	51.29	0.14%
**Boiler part**			
Heat transfer to cycle part (MW)	1952.51	1949.63	−0.15%
Heat transfer of flue gas cooler (MW)	58.83	58.62	−0.35%
Heat loss of exhaust flue gas (MW)	118.02	117.58	−0.38%
Mass flow of coal (t/h)	317.54	315.85	−0.53%
Efficiency of boiler (%)	94.43	94.79	0.38%

**Table 2:** The properties of selected coal [29].

C_ar_ (%)	H_ar_ (%)	O_ar_ (%)	S_ar_ (%)	N_ar_ (%)	M_ar_ (%)	A_ar_ (%)	LHV (kJ/kg)
61.70	3.67	8.56	0.60	1.12	15.55	8.80	23,442

**Table 3:** Energy equilibrium equations of main components.

Components	Energy Equilibrium Equations ^a^
Boiler heating surface	${\dot{m}}_{\mathrm{fg}}\cdot (h_{\mathrm{fg},\mathrm{in}}-h_{\mathrm{fg},\mathrm{out}})={\dot{m}}_{{\mathrm{SCO}}_{2}}\cdot (h_{{\mathrm{SCO}}_{2},\mathrm{out}}-h_{{\mathrm{SCO}}_{2},\mathrm{in}})$
Recuperator	${\dot{m}}_{\mathrm{hot}}\cdot (h_{\mathrm{hot},\mathrm{in}}-h_{\mathrm{hot},\mathrm{out}})={\dot{m}}_{\mathrm{cold}}\cdot (h_{\mathrm{cold},\mathrm{out}}-h_{\mathrm{cold},\mathrm{in}})$
Turbine	${\dot{W}}_{t}={\dot{m}}_{t}\cdot (h_{t,\mathrm{in}}-h_{t,\mathrm{out}})$
Compressor	${\dot{W}}_{c}={\dot{m}}_{c}\cdot (h_{c,\mathrm{out}}-h_{c,\mathrm{in}})$
Precooler	${\dot{Q}}_{p}={\dot{m}}_{p}\cdot (h_{p,\mathrm{in}}-h_{p,\mathrm{out}})$

^a^ *h*—enthalpy, $\dot{m}$—mass flow rate, $\dot{W}$—power, $\dot{Q}$—heat rate. Subscript: fg—flue gas, t—turbine, c—compressor, p—precooler, in—inlet, out—outlet, hot—hot side, cold—cold side.

**Table 4:** Input parameters of typical system models.

Parameters	Values
Maximum temperature of system (*t*_max_)	600 °C ^a^
Maximum pressure of system (*p*_max_)	30 MPa ^a^
Reheat pressure (*p*_rh_)	16 MPa
Minimum pressure of system (*p*_min_)	7.6 MPa ^b^
Minimum temperature of system	32 °C ^a^
Compressor isentropic efficiency	0.89 ^b^
Turbine isentropic efficiency	0.93 ^a^
Generator efficiency	0.99 ^a^
Pinch temperature difference of recuperator	10 °C ^c^
Pressure drop in components except for boiler	0.1 MPa ^b^
Pressure drop in superheat part of boiler	0.6 MPa
Pressure drop in reheat part of boiler	0.25 MPa
Pressure drop in tail heater of boiler	0.1 MPa
Excess air coefficient	1.2 ^c^
Split ratio to tail heater	0.1 ^d^
Hot air temperature	340 °C
Exhaust temperature of flue gas	120 °C
Output electric power of system	300 MW

^a^ Ref. [38]. ^b^ Ref. [39]. ^c^ Ref. [29]. ^d^ Ref. [40].

**Table 5:** Added input parameters owing to the improved schemes.

Schemes	Parameters	Values
Double reheat	Inlet temperature of low-pressure turbine 2	600 °C ^a^
	Inlet pressure of low-pressure turbine	20 MP ^a^
	Inlet pressure of low-pressure turbine 2	13 MP ^a^
	Pressure drop in double reheater of boiler	0.2 MP ^a^
Double recompression	Second split ratio	0.15
Intercooling	Inlet pressure of intercooler (*p*_ic_)	9.3 MP ^a^
	Inlet temperature of intercooler	32 °C ^a^

^a^ Ref. [38].

**Table 6:** The values of economic parameters.

Symbols	Economic Parameters	Values
*n*	system economic lifetime	20 year ^a^
*τ*	annual operation hour	8000 h/year ^a^
*i*_e_	annual effective interest rate	0.10 ^a^
*r*_n,OMC_	annual nominal escalation rate of *OMC*	0.025 ^a^
*r*_n,FC_	annual nominal escalation rate of *FC*	0.025 ^b^
*ψ*	relation coefficient	1.3608 ^c^
*c*_coal_	coal price per unit of heat	4.09 USD/GJ ^d^
*φ*_fix_	fixed cost coefficient	0.015 ^e^
*φ*_var_	variable cost coefficient	1.65 USD/MW ^e^

^a^ Ref. [44]. ^b^ Ref. [45]. ^c^ Ref. [46]. ^d^ Ref. [47]. ^e^ Ref. [5].

**Table 7:** The values of characterization factor (*CF*).

Environmental Impact Categories	Units	Pollutants	*CF*
GWP	kg CO_2_-eq/kg	CO_2_	1 ^a^
AP	kg SO_2_-eq/kg	SO_2_	1 ^a^
		NO_x_	0.7 ^a^
HTP	kg 1,4-DB-eq/kg	SO_2_	0.096 ^a^
		NO_x_	1.2 ^a^
DP	kg dust-eq/kg	dust	1 ^b^

^a^ Ref. [53]. ^b^ Ref. [28].

**Table 8:** The values of *CEI*_90_ and *WF*.

Environmental Impact Categories	*CEI*_90_	*WF*
GWP	8700	0.83
AP	36	0.73
HTP	24.65	0.73
DP	18	0.61

**Table 9:** The values of the gamultiobj parameters.

Parameters	Section 4.2	Section 4.4
Population size	100	100
*MaxGen*	1200	200
Pareto fraction	0.35 (*Gen* ≤ 1000)	0.35 (*Gen* ≤ 150)
	1 (*Gen* > 1000)	1 (*Gen* > 150)
Others	default	default

**Table 10:** Comparison of system efficiency (*η*_sys_) and purchased equipment cost (*PEC*) for four typical layouts.

Items	Case A	Case B	Case C	Case D
**Efficiency (%)**				
*η*_sys_	46.20	47.85	47.19	47.89
***PEC*** ** of identical components (USD)**				
Boiler	1.43 × 10^8^	1.39 × 10^8^	1.40 × 10^8^	1.39 × 10^8^
High-pressure turbine (HT)	5.67 × 10^6^	5.77 × 10^6^	5.55 × 10^6^	5.78 × 10^6^
Low-pressure turbine (LT)	4.76 × 10^6^	4.85 × 10^6^	4.64 × 10^6^	4.84 × 10^6^
Main compressor (MC)	8.36 × 10^6^	8.13 × 10^6^	8.22 × 10^6^	8.12 × 10^6^
Recompressor (RC)	8.14 × 10^6^	9.14 × 10^6^	9.25 × 10^6^	9.13 × 10^6^
High-temperature recuperator (HTR)	2.87 × 10^7^	4.66 × 10^7^	2.78 × 10^7^	2.72 × 10^7^
Low-temperature recuperator (LTR)	3.39 × 10^7^	3.47 × 10^7^	3.55 × 10^7^	3.47 × 10^7^
Precooler (PC)	5.26 × 10^6^	4.88 × 10^6^	5.03 × 10^6^	4.87 × 10^6^
Generator	2.46 × 10^6^	2.46 × 10^6^	2.46 × 10^6^	2.46 × 10^6^
***PEC*** ** of added components (USD)**				
Split flow recuperator (SFR)	-	-	2.57 × 10^6^	5.18 × 10^6^
Split flow turbine (SFT)	-	-	1.76 × 10^6^	-

**Table 11:** Comparison of system efficiency (*η*_sys_) and purchased equipment cost (*PEC*) for three improved layouts.

Items	Case D	Case D1	Case D2	Case D3
**Efficiency (%)**				
*η*_sys_	47.89	48.20	48.60	48.32
***PEC*** ** of identical components (USD)**				
Boiler	1.39 × 10^8^	1.38 × 10^8^	1.37 × 10^8^	1.38 × 10^8^
HT	5.78 × 10^6^	4.51 × 10^6^	6.21 × 10^6^	5.64 × 10^6^
LT	4.84 × 10^6^	4.01 × 10^6^	5.19 × 10^6^	4.72 × 10^6^
MC	8.12 × 10^6^	8.08 × 10^6^	8.03 × 10^6^	2.32 × 10^6^
RC	9.13 × 10^6^	9.09 × 10^6^	9.03 × 10^6^	8.62 × 10^6^
HTR	2.72 × 10^7^	2.85 × 10^7^	1.64 × 10^7^	2.61 × 10^7^
LTR	3.47 × 10^7^	3.43 × 10^7^	3.39 × 10^7^	2.49 × 10^7^
PC	4.87 × 10^6^	4.80 × 10^6^	4.71 × 10^6^	5.01 × 10^6^
Generator	2.46 × 10^6^	2.46 × 10^6^	2.46 × 10^6^	2.46 × 10^6^
SFR	5.18 × 10^6^	5.43 × 10^6^	3.13 × 10^6^	4.98 × 10^6^
***PEC*** ** of added components (USD)**				
LT2	-	3.60 × 10^6^	-	-
Medium-temperature recuperator (MTR)	-	-	6.27 × 10^7^	-
Double recompressor (DRC) or MC2	-	-	8.50 × 10^6^	7.25 × 10^6^
Intercooler (IC)	-	-	-	2.22 × 10^6^

**Table 12:** Spearman correlation coefficient among seven parameters.

Rank	Parameters	Values	Rank	Parameters	Values
1st	*UEI* and *LCOE*	−1.000	12th	*p*_ic_ and *LCOE*	0.909
2nd	*t*_max_ and *LCOE*	0.997	12th	*p*_ic_ and *UEI*	−0.909
2nd	*t*_max_ and *UEI*	−0.997	14th	*t*_max_ and *p*_ic_	0.903
4th	*p*_max_ and *LCOE*	0.979	15th	*p*_max_ and *p*_ic_	0.901
4th	*p*_max_ and *UEI*	−0.979	16th	*p*_rh_ and *p*_min_	0.888
6th	*t*_max_ and *p*_max_	0.966	17th	*p*_max_ and *p*_min_	0.885
7th	*p*_rh_ and *LCOE*	0.947	18th	*p*_min_ and *p*_ic_	0.885
7th	*p*_rh_ and *UEI*	−0.947	19th	*p*_min_ and *LCOE*	0.879
9th	*t*_max_ and *p*_rh_	0.943	19th	*p*_min_ and *UEI*	−0.879
10th	*p*_max_ and *p*_rh_	0.932	21st	*t*_max_ and *p*_min_	0.870
11th	*p*_rh_ and *p*_ic_	0.919			

**Table 13:** Comparison of three optimal points.

Points	*t*_max_ (°C)	*p*_max_ (MPa)	*p*_rh_ (MPa)	*p*_min_ (MPa)	*p*_ic_ (MPa)	*UEI* (kp-eq)	*LCOE* (USD/MWh)
DOP	635.3	30.08	15.95	7.602	9.216	213.8	58.29
ENOP	700.0	40.00	18.26	7.639	10.68	200.7	70.82
ECOP	591.1	25.35	15.74	7.596	8.738	225.8	56.37

## Data Availability

Not applicable.

## Nomenclature

*AE*	annual emissions (kg/year)
*c*_coal_	coal price per unit of heat (USD/GJ)
*CEI*	environmental impact per capita (kg pollutant-eq/year·p-eq)
*CELF*	constant escalation levelization factor
*CF*	characterization factor
*CLC*	closeness coefficient
*CP*	characteristic parameter
*CRF*	capital recovery factor
*d*	Euclidean distance
*EI*	environmental impact (kg pollutant-eq/year)
*f_p_*	pressure correction coefficient
*f_t_*	temperature correction coefficient
*Gen*	generation number
*h*	enthalpy (kJ/kg)
*i*_e_	annual effective interest rate
*LCOE*	levelized cost of electricity (USD/kWh)
$\dot{m}$	mass flow rate (kg/s)
*MaxGen*	maximum generation number
*MF*	mass fraction
*n*	system economic lifetime (year)
*NEI*	normalized environmental impact (p-eq)
*p*	pressure (MPa)
*PEC*	purchased equipment cost (USD)
$\dot{Q}$	heat rate (kW)
*r*_n_	annual nominal escalation rate
*t*	temperature (°C)
*TCI*	total capital investment (USD)
*UEI*	ultimate environmental impact (p-eq)
$\dot{W}$	power (kW)
*WF*	weight factor
**Abbreviations**	
AP	acidification potential
BP	British Petroleum
CC	carrying charges
DOP	decision optimal point
DP	dust pollution potential
DRC	double recompressor
DRH	double reheater
ECOP	economic optimal point
ENOP	environmental optimal point
FC	fuel costs
GWP	global warming potential
HT	high-pressure turbine
HTP	human toxicity potential
HTR	high-temperature recuperator
IC	intercooler
LHV	low heat value
LT	low-pressure turbine
LTR	low-temperature recuperator
MC	main compressor
MTR	medium-temperature recuperator
NSGA-II	fast elitist non-dominated sorting genetic algorithm
OMC	operating and maintenance costs
PC	precooler
RC	recompressor
RHP	reheat part
SC	system costs
SCO_2_	supercritical carbon dioxide
SCPG	SCO_2_ coal-fired power generation
SFR	split flow recuperator
SFT	split flow turbine
SHP	superheat part
TH	tail heater
TOPSIS	technique for order preference by similarity to an ideal solution
TRR	total revenue requirement
**Greek letters**	
Δ*t*	pinch temperature difference (°C)
*η*_sys_	system efficiency (%)
*τ*	annual operation hour (h/year)
*ψ*	relation coefficient
*φ*_fix_	fixed cost coefficient
*φ*_var_	variable cost coefficient (USD/MWh)
**Subscripts**	
0	the first year
c	compressor
cold	cold side
ele	electricity
ex	exhaust
fg	flue gas
hot	hot side
*i*	*i*th environmental impact category or *i*th candidate
ic	intercooling
in	inlet
*j*	*j*th pollutant or *j*th indicator
*l*	levelized
max	maximum
min	minimum
out	outlet
p	precooler
r	recuperator
rh	reheat
t	turbine
tot	total
